# Evaluation of chloroplast genome annotation tools and application to analysis of the evolution of coffee species

**DOI:** 10.1371/journal.pone.0216347

**Published:** 2019-06-12

**Authors:** Christophe Guyeux, Jean-Claude Charr, Hue T. M. Tran, Agnelo Furtado, Robert J. Henry, Dominique Crouzillat, Romain Guyot, Perla Hamon

**Affiliations:** 1 Femto-ST Institute, UMR 6174 CNRS, Université de Bourgogne Franche-Comté, Besançon, France; 2 Queensland Alliance for Agriculture and Food Innovation, University of Queensland, Brisbane, QLD, Australia; 3 Nestlé Centre Tours, Tours, France; 4 Institut de Recherche pour le Développement, UMR IPME, CIRAD, Université de Montpellier, Montpellier, France; 5 Department of Electronics and Automatization, Universidad Autónoma de Manizales, Manizales, Colombia; 6 Institut de Recherche pour le Développement, UMR DIADE, Université de Montpellier, Montpellier, France; National Cheng Kung University, TAIWAN

## Abstract

Chloroplast sequences are widely used for phylogenetic analysis due to their high degree of conservation in plants. Whole chloroplast genomes can now be readily obtained for plant species using new sequencing methods, giving invaluable data for plant evolution However new annotation methods are required for the efficient analysis of this data to deliver high quality phylogenetic analyses. In this study, the two main tools for chloroplast genome annotation were compared. More consistent detection and annotation of genes were produced with GeSeq when compared to the currently used Dogma. This suggests that the annotation of most of the previously annotated chloroplast genomes should now be updated. GeSeq was applied to species related to coffee, including 16 species of the *Coffea* and *Psilanthus* genera to reconstruct the ancestral chloroplast genomes and to evaluate their phylogenetic relationships. Eight genes in the plant chloroplast pan genome (consisting of 92 genes) were always absent in the coffee species analyzed. Notably, the two main cultivated coffee species (i.e. Arabica and Robusta) did not group into the same clade and differ in their pattern of gene evolution. While Arabica coffee (*Coffea arabica*) belongs to the *Coffea* genus, Robusta coffee (*Coffea canephora*) is associated with the *Psilanthus* genus. A more extensive survey of related species is required to determine if this is a unique attribute of Robusta coffee or a more widespread feature of coffee tree species.

## Introduction

In plants, the chloroplast genome is circular with generally a quadripartite structure including two copies of a large inverted repeat (IR) separated by large and small single-copy regions (LSC and SSC, respectively). This organization is generally highly conserved [1, 2, 3] with some exceptions reported such as loss of inverted repeats in gymnosperms [4].

For several decades, plastid sequences polymorphisms have been used to assess with more or less accuracy maternal phylogenetic relationships between orders, families, tribes, genera and species. The development of low-cost high-throughput sequencing has permitted huge amounts of raw data of both nuclear and plastid sequences to be obtained. In consequence complete chloroplast sequences are more and more widely used and provide invaluable data for doubtful phylogenies, plant evolution [5, 6], barcoding and for a better understanding of patterns of gene loss or of adaptive changes in relation to photosynthesis [7]. In terms of gene content and order, conservation is observed for the majority of genes. Therefore, among the 126 genes found in a large set of angiosperm chloroplast genomes (272 species, including gymnosperms, eudicots, monocots, and basal angiosperms), 106 were shared by 245 species (90%) and 13 were present in only 27 species (9.9%) [8].

This result was congruent with earlier findings for a collection of 99 chloroplast genomes of photosynthetic Eucaryotic lineages [9] that asked: which are the genes shared by all the species of a collection (‘core genome’, 106 genes in [8]) and conversely, which are all the genes found considering the species of the collection (‘pan genome’, the 126 genes in [8])?

However, accurate comparative genomics could be obscured due to the use of inappropriate annotation methods [10], including DOGMA [11] and CpGAVAS that may be unsuitable for the annotation of more than one genome per analysis [13] and are considered to be out-of-date, being widely used [8, 12] despite the availability of new annotation methods that have been recently developed [14, 15]. The objective of this study is to demonstrate the importance of an accurate chloroplast annotation, using a dataset from the *Coffea* genus.

Worldwide coffee (*Coffea* spp.) production for 2017 was 158 million 60-kg bags including Arabica (*Coffea arabica* L., 97.2 Mbags) and Robusta (*Coffea canephora* Pierre, 61.4 Mbags), worth a total of 25.4 billion U.S. dollars [16]. Underscoring the importance of coffee to humanity, the 60 countries producing coffee employ about 100 million people. However, despite the socio-economic importance of these species, research on them remains mainly applied. Few more ambitious studies devoted to increasing fundamental knowledge of aspects of coffee biology or related to topics such as genome evolution [17], speciation [18] and adaption to various biotic and abiotic stresses [19] have been conducted to date. One of the unresolved issues of coffee evolution remains the clear determination of their relationships permitting the classification of these species into one or two genera. Until 2011, coffee trees were classified in the *Coffea* genus, as a close relative to the *Psilanthus* genus in the Rubiaceae family [20]. The separation of these two genera was estimated to be 12 million years ago [18]. Their distinction was mainly based on flower morphology and on their geographical distribution. Both genera occur in Africa but *Coffea* was absent in Asia while *Psilanthus* was absent in Western Indian Ocean Islands. They also differed by their main mating system. *Coffea* is mainly self-sterile except for two diploids [21, 22] and the tetraploid *C*. *arabica* while *Psilanthus* is self-fertile. However, despite a lack of support for the phylogeny, *Psilanthus* was subsumed into *Coffea* [23] leading to confusion in species names and relationships.

The molecular phylogeny of coffee species based upon 28,800 nuclear SNPs suggested clear relationships between species but failed to provide a definitive answer to the botanical classification since one *Coffea* species (*C*. *rhamnifolia*) was nested in the *Psilanthus* clade [18]. A chloroplast genome based evolutionary history of coffee species (broad sense) may provide more information and help to resolve the relationships between *Coffea* and *Psilanthus*.

In this study several questions have been addressed: Would maternal phylogenetic species relationships based upon the whole chloroplast sequences give similar topology to an update of the last published nuclear phylogeny [18]? Could a methodology be defined for accurate annotation of chloroplast genomes? What could be learned in terms of gene content, order organization and reconstruction of the ancestral chloroplast genomes by applying this to coffee chloroplast genomes?

To answer these questions, a set of chloroplast genomes assembled from coffee species was used first to assess the maternal phylogenetic relationships. Two tools for chloroplast annotation [11, 14] were compared for their accuracy. The better quality annotations were retained and used for comparative genomics (gene content and structure changes such as duplication, inversion, etc.) and ancestral chloroplast genome reconstruction.

## Material and methods

### Data collection

Two sets of complete chloroplastic sequences have been considered in this study. Firstly, a Python script has been written to automatically download all (non annotated) fasta files of complete chloroplast genomes (Cp genomes) available on the NCBI, leading to 2,112 sequences. This set contains only two coffee species, namely *Coffea canephora* (accession KU500324) and *Coffea arabica* (accession NC 008535), and no *Psilanthus* was available. Chloroplast genomes from *Coffea* and *Psilanthus* were obtained as follows. The DNA of 16 diploid coffee species (Table 1), representatives of the biogeographic group as previously defined [18], were sequenced using Illumina technology. Chloroplast sequences were filtered and assembled. Among the 16 species, twelve were provided by the *Coffea* 13-Genomes consortium [24]. *Psilanthus brassii* was collected from the Australian Tropical Herbarium, a *C*. *canephora* (accession DH-200-94) sequence was mined from the *C*. *canephora* genome project [17]. This sequenced data needed to be assembled, and the procedures applied to do this are described below. *C*. *arabica* (accession NC_008535) sequence was retrieved from NCBI.

**Table 1:** Species considered in this study for chloroplast genomes analyses. Herbarium codes after Holmgren et al. (1990). Germplasm source BR (Botanic Gardens of Belgium), BRC (Biological Resources Center, Reunion), CBI (Coffee Board of India), K (Royal Botanic Gardens, Kew (UK), KCRS (Kianjavato Coffee Research Station (Madagascar), P (Natural History Museum, Paris (France)).

Species name	Plant, voucher herbarium code	Country of origin [African sub-region]	Germplasm collection source
***Psilanthus***			
*P*. *benghalensis var bababudanii (Sivar*., *Biju & P*.*Mathew) A*.*P*.*Davis*	PBT1 (CCRI)	India	CBI
*P*. *benghalensis (Heyne ex J*.*A*. *Schult*.*) Leroy*	PBT5 (CCRI)	India	CBI
*P*. *brassii (J*.*-F*.*Leroy) A*.*P*.*Davis*	D. Crayn 1196 (CNS)	Australia	CNS
*P*. *ebracteolatus Hiern*	PSI11 (K,P)	Ivory Coast	BRC
*P*. *horsfieldianus (Miq*.*) J*.*-F*. *Leroy*	HOR (K)	Indonesia	ICRI
*P*. *mannii Hook*.*f*.	2003 1365–45 (BR)	Cameroon	BR
***Coffea***			
*C*. *arabica L*.	NA	NA	NC_008535
*C*. *canephora Pierre ex A*.*Froehner*	DH200-94	Democratic Republic of Congo	BRC
*C*. *humblotiana Baill*.	BM19/20 (K, MO, TAN)	Comoros	BRC
*C*. *humilis A*.*Chev*.	G57 (K)	Ivory Coast	BRC
*C*. *macrocarpa A*.*Rich*.	PET (P, K)	Mauritius	BRC
*C*. *dolichophylla J*.*-F*.*Leroy*	A.206 (P)	Madagascar	KCRS
*C*. *pseudozanguebariae Bridson*	H53 (K)	Kenya	BRC
*C*. *racemosa Lour*.	IB62 (K)	Mozambique	BRC
*C*. *stenophylla G*.*Don*.	FB55 (K)	Ivory Coast	BRC
*C*. *tetragona Jum*. *& H*.*Perrier*	A.252 (K, MO, TAN)	Madagascar	KCRS

### Plastid genome reconstruction

All steps, unless otherwise indicated, were carried out using the CLC Genomics Workbench (CLC-GWB) software (CLC Genomics Workbench 7.0.4, http://www.clcbio.com). Raw reads were imported into CLC-GWB. In addition, a sequence of the *C*. *arabica* plastid genome from NCBI (accession NC 008535) was imported to CLC and used as a reference sequence [25]. Raw reads were subjected to Quality Control (QC) analysis, which was used as a guide for trimming the reads. Low-quality paired-end sequence reads were trimmed using default parameters. The quality score limit was set to 0.05 (corresponding to PHRED quality value > 15) and the minimum number of nucleotides in reads was 15 bp. These reads were used for the assembly of the plastid genomic sequence using two approaches: reference-guided mapping assembly and de novo assembly. For reference-guided mapping assembly, the trimmed reads were subjected to read mapping using *C*. *arabica* as the reference sequence (parameter settings for read mappings: no masking mode, mismatch cost: 2, insertion cost: 3, deletion cost: 3, length fraction: 0.5, similarity fraction: 0.5, no global alignment, auto-detect paired distances: yes, non-species match handling: map randomly, output mode: create stand-alone read mappings). In addition, the consensus sequence derived from the mapping step was used as a reference sequence. Indel structural variant analysis was performed based on the mapping files with p-value threshold of 0.0001. Predicted indel structural variants were used as a guidance-variant track for local re-alignment to create the standalone mapping, and the consensus sequence was then used as the mapping-derived assembled genome sequence. Trimmed reads were then mapped to this genome to obtain the mapping-Cp-mapping file. For de novo assembly, common settings were applied for all species (mapping mode: create simple contig sequence (fast), perform scaffolding: no, auto-detect paired distances: yes, colorspace alignment: no, guidance only reads: no, min distance: 180, maximum distance: 250), except that various combinations of settings for word sizes (20, 25 and 40) and bubble sizes (50, 70, 80, 100 and 120) as well as minimum contig lengths (800 and 2000) were executed. The best contigs were chosen on the basis of covering the entire plastid genome with no gaps with contigs with the highest N50. The contigs were then subjected to BLAST analysis (performed within CLC) against the *C*. *arabica* plastid genome as the reference for the selection of long contigs (parameter settings: program: BLASTN, match 2 mismatch 3 gap cost existence 5 gap cost extension 2, expectation value (5.0), word size: 11, no mask lower case, filter low complexity: yes, maximum number of hits: 250, number of threads: 1). The selected contigs were then aligned to the *C*. *arabica* plastid reference sequence using Clone Manager 9 (SciEd, USA) (parameter settings: "Global DNA alignment" (align all sequences against a reference sequence, with the alignment spanning the entire length of sequences specified)). Scoring matrix: standard linear (mismatch penalty: 2, open gap penalty: 4, extend gap penalty: 1) was used to determine correct sequence orientation and ensure complete coverage of the reference plastid sequence. The selected and correctly oriented contigs were manually updated using the Update Contig tool in the CLC Genomics Workbench. The updated contigs were aligned back to the reference sequence, the overlaps were determined, and stitching of the contigs at the overlaps was undertaken to obtain a de novo-derived plastid genome sequence. The trimmed reads were mapped to this genome sequence to obtain a corresponding de novo mapping file. The de novo-derived and mapping-derived plastid genome sequences were aligned to determine any discrepancies between the two assemblies. Any discrepancies observed were manually curated by observing reads mapped at corresponding nucleotide positions in the reference-based and de novo mapping files. In some cases, (*C*. *racemosa*, *C*. *stenophylla*, and *P*. *brassii*), sufficient contigs were obtained to generate whole plastid genome sequences after a few de novo assemblies. However, the assembled plastid genomes of other species had gaps due to missing contigs. In such cases, more de novo assemblies were generated using different settings of word and bubble sizes, and using contigs from several de novo assembly results to fill the gaps. If after running the first local realignment, the first two consensus genomes from reference guided mapping assembly and the consensus from de novo assembly still had numerous discrepancies of more than 50 (both SNPs and indels; indels were counted as 1 discrepancy), additional mapping of trimmed reads using the consensus sequence from the preceding mapping steps as a reference sequence was performed, followed by local realignments.

### Phylogenetic study

A first nuclear phylogeny was computed on the 101 nuclear sequences of coffee species obtained from [18] (81 samples and new unpublished data for 15 additional samples, see S1 Table. The alignment was performed using Muscle [26] (28,800 SNPs). The phylogenetic reconstruction was done using RAxML [27] (General Time Reversible nucleotide substitution model with gamma distributed rate variation among sites and Felsenstein's bootstraps) with five outgroups (*Bertiera iturensis*, *Belonophora coriacea*, *Argocoffeopsis eketensis*, *Calycosiphonia spathicalyx* and *Tricalysia congesta*, all Rubiaceae members) and the tree visualized with FigTree ver. 1.3.1 [28]. The tree obtained was cross-validated using Mr Bayes [29]. Furthermore, a phylogenetic network was plotted (NeighborNet method, as implemented in SplitsTree4 [30]) to investigate horizontal transfers between species. A maternal phylogeny was performed using the 16 Cp sequences obtained from the *Coffea* and *Psilanthus* samples listed in Table 1 plus sequences from *Emmenopterys henryi* (validated with *Solanum lycopersicum* as outgroups) obtained from NCBI (accession numbers NC 036300.1 for *E*. *henryi* and NC 007898.3 for *S*. *lycopersicum*). The full assembled sequences were aligned using ClustalW ver. 2.1 [31], while RAxML was used to infer the phylogenetic tree (GTR substitution model with gamma distributed rate variation among sites, Felsenstein’s bootstraps) visualization with ETE3 [32].

### Chloroplast annotations

Three approaches to chloroplast annotation were investigated: (i) use of annotation websites specific to organelle genomes, Dogma [11] or GeSeq [14]; (ii) starting from a reference coffee chloroplast annotation (*C*. *arabica* accession NC 008535: this is of particular interest because of its economic and social importance, leading to a well-studied genome) or a related species annotation translated to new chloroplasts genomes (using BLAST [33] or a specific tool to translate annotations); and (iii) to proceed as in [34]. Briefly, a large collection of annotated Cp coding sequences were downloaded on NCBI. Then, local BLAST was used to predict gene locations. The sequences were clustered with the small clusters (size 1 and 2) discarded (probably an annotation problem). For each cluster, the resulting consensus was blasted against the NCBI database to assign a gene name.

The reconstruction of ancestral genomes was based on the gene content. Firstly, the ancestral ordering at each internal node of the tree (showing possible insertions, deletions, or inversions of genes) was considered, and then the ancestral sequences of each gene, and all intergenic regions.

### Mutation investigation

Indels and gaps along the Cp genomes were investigated as follows: the number of polymorphic nucleotides, the number of indels and gaps, the average size and standard deviation of the gaps, the proportion (percentage) of positions with indels, polymorphisms, or gaps. The question of whether these variations were evenly distributed in the genomes or if there were areas more conducive to this (recombination hot spots), was investigated. The number of polymorphic columns in the alignment (without the outgroup) was evaluated, taking care to spread the columns for which the polymorphism was only carried by the outgroup: one InDel, for instance was a column of the alignment that contain a symbol '-' such that its two bordering columns do not contain this symbol. Conversely, a gap in the alignment was a hole of length > 1. Particular attention was given to the mutation comparison between *Coffea* and *Psilanthus* on the one hand, and between genes versus non-coding sequences on the other hand. The distribution of these mutations was evaluated (for example, were some genes more affected than others?) depending on the possibly silent nature of such mutations.

### Ancestral genome reconstruction

The well-supported phylogenetic tree and the good annotation of the genomes, produced in the previous phases of this study, made it was possible to use the latter as a backbone structure in the reconstruction of ancestral chloroplast genomes. The evolutionary relationship was reconstructed in the form of a binary tree, in which the species were descendants placed at the leaves, while the internal nodes were extinct ancestors connected by edges. Ancestral genome reconstruction can be achieved by mixing state-of-the-art algorithms and manual investigations if the genomes being considered do not diverge too much. The reconstruction of ancestors of both *Coffea* and *Psilanthus* genera was carried out as follows. Firstly, the genomes were separated into two clades: the *Coffea* and the *Psilanthus* as in the nuclear phylogenic tree reconstructed with RAxML. However, the plastid *Psilanthus* clade also included *Coffea canephora*. The genomes of each clade were aligned using clustalw [31] and the phylogenic tree for each clade was recomputed again with RAxML with GTR Gamma model as advised by JModelTest 2.0. Following this, the internal nodes of the trees were renamed using the bio-python package [35], and the tree model (including the substitution rate matrix, branch lengths, and estimates of the nucleotide equilibrium frequencies), were computed with PhyloFit (a program in Phast [36]) using the maximum likelihood method [37]. Finally, the ancestral sequences were reconstructed by Prequel (a program in Phast) that computes the marginal probability distributions for each base in the ancestral nodes in the phylogenic tree using the tree model and the sum-product algorithm [38]. The reconstructed ancestors were then verified with Ad hoc algorithms designed to deal with low marginal probability distributions. These algorithms asked for human intervention if some cases cannot be resolved automatically [39].

## Results

### GeSeq, an efficient tool for chloroplast genome annotation

**Fig 1:**
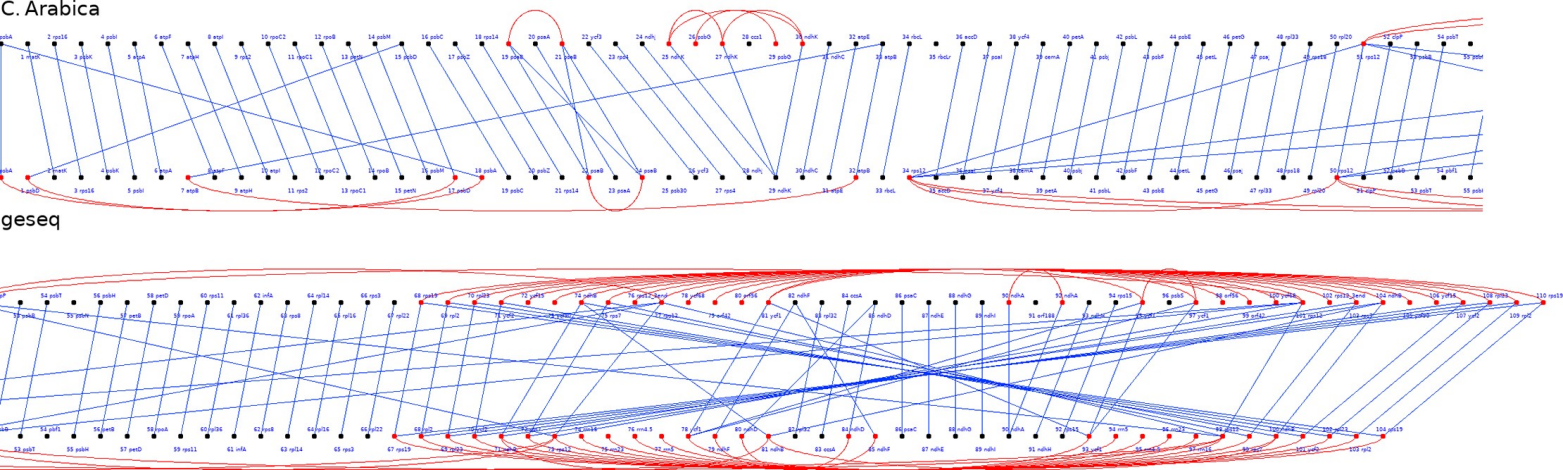
Annotation differences from Dogma and GeSeq using *C*. *arabica* Cp genome as study case. The Cp genome, split into two parts, is described as an ordered list of predicted coding sequences, leading to two black dotted lines for each part, surrounded by gene names written in blue. In each part, the upper line entitled *C*. *arabica* corresponds to the Dogma annotations, while the lower line is for GeSeq. A red edge indicates paralogous genes within a given genome, while a blue line is drawn when predicted sequences are similar between the two annotations.

Two tools for chloroplast annotation, namely Dogma and GeSeq, were compared for their efficiency and accuracy based upon the analysis of a set of 16 coffee trees genomes. Our results showed that they do not provide compatible annotations, as depicted in Fig 1 taking *Coffea arabica* as example. The gene names differed between the two annotations (e.g. *psbN* versus *pbf1*) and GeSeq predicted genes that Dogma failed (cf. *psbD* at position 1 and *isba* at position 18), but the converse is also true (*ccs1* at position 28 in Dogma annotated genome missing with GeSeq annotation). The lack of stability in gene detection was mainly found with Dogma. This is signaled for instance by the *ycf62* gene: it is found by Dogma in position 6918–6971 in *P*. *benghalensis* var bababudanii, and it is absent from *P*. *brassii*, always according to this annotation tool. However, a simple BLAST returns exactly the same sequence in *P*. *brassii*, in position 6870–6923. Either this sequence is a coding one or not, but the same decision should be taken when two very similar sequences are found in two close genomes.

**Fig 2:**
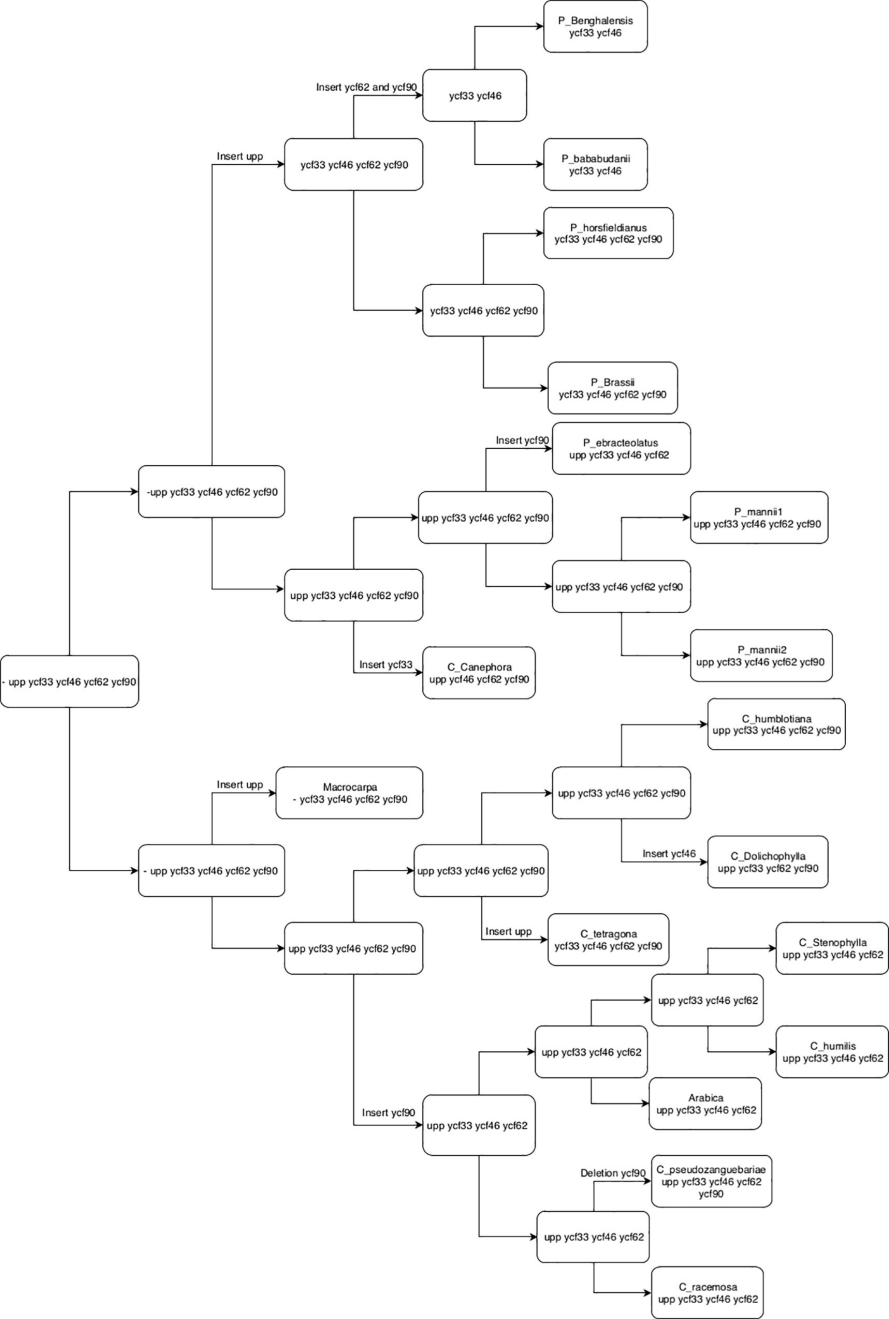
Evolution of gene content based on Dogma annotations. Each node contains the genome pan genes that are missing in the species under consideration (at the level of leaves, for species currently living, and at the level of internal nodes, concerning their ancestors). Gene insertion or deletion events are indicated at the branch level.

Another illustration of this lack of stability leading to incompatible situations is the deduced evolution of gene contents based on the annotations provided, as depicted in Fig 2. This figure focuses on genes that are outside the core genome of Dogma, and it deduces the most probable evolution (insertion or deletion) of gene content according to a parsimonious approach. Such a scenario leads to events that are unlikely or even not parsimonious, such as the insertion of a gene followed by its deletion, or the independent insertion of the same gene in multiple places in the tree: each time, a detection error in one or more genomes makes it possible to provide a more conclusive answer. Furthermore, Dogma predicted various pseudogenes that are detected only in reduced variable subsets of chloroplasts (e.g. *ycf62*: an unreviewed protein inferred from homology, whose annotation score is 2/5 according to UniProt [40]). These putative pseudogenes are not detected by Geseq, leading to more coherence in the gene content evolution that can be deduced using these annotations. As an exception to the use of GeSeq, all the other possible annotation approaches are based, in some way or another, on Dogma. Finally, Dogma annotation leads to predicted coding sequences that are not sufficiently reliable. Conversely, GeSeq succeeded in recovering genes of well annotated plant genomes. We thus decided to use only GeSeq for the annotation step in this study.

### Nuclear coffee phylogenenetic relationships

**Fig 3:**
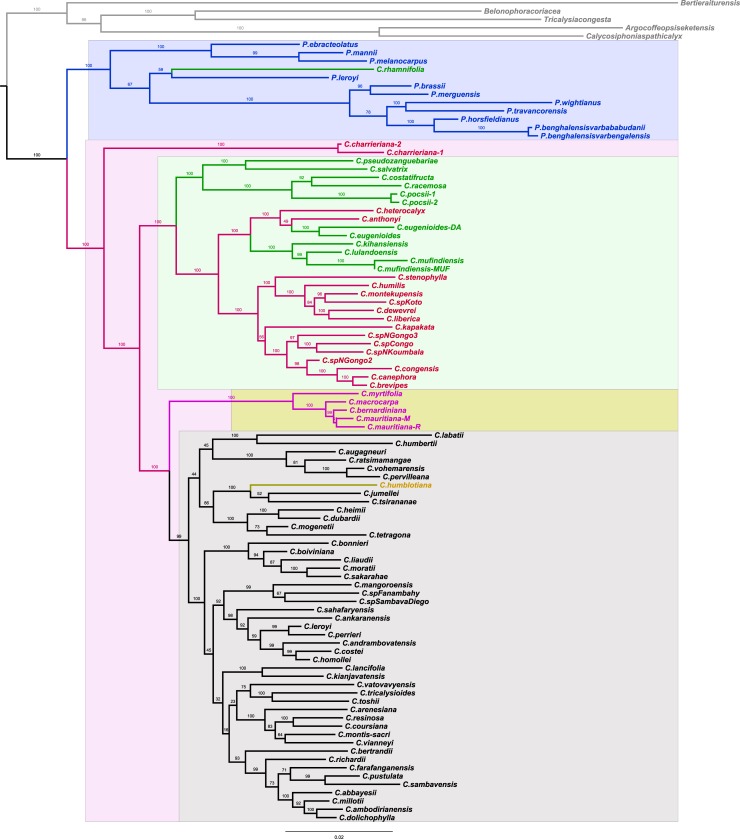
Maximum likelihood nuclear phylogeny of coffee trees. The molecular tree was based on 22,800 SNPs as described in [18] and a larger set of species (see S1 Table). Branch lengths are proportional to inferred nucleotide substitutions. The values at the nodes indicate the bootstrap support of each branch in percent. The main clades are shaded, i.e., blue for *Psilanthus*, pink for *Coffea* and among this latter group the main geographic areas are shaded pale green for Africa, yellow for Mascarenes and pale grey for Madagascar and Comoros (yellowish branch).

Nuclear phylogenetic analysis using maximum likelihood, based upon Hamon’s et al data [18] and additional samples, led to two separated clades for *Psilanthus* (pale violet shadow box) and *Coffea* (pale pink shadow box) genera. Inside the latter, three separated clades appeared, depending on the geographic spread of the *Coffea* species: Africa (pale green shadow box), Mauritius—Reunion (pale yellow shadow box), or Madagascar (light grey shadow box), see Fig 3. Compared to previous results [18], additional samples were positioned in concordance with their native area. The African *P*. *melanocarpus* was in the African *Psilanthus* clade while the Australian *P*. *brassii* and the Thai *P*. *merguensis* fell in the Asian *Psilanthus* clade. Similarly, the Tanzanian *C*. *mufindiensis* was close to the East African clade comprising *C*. *kihansiensis* and *C*. *lulandoensis* while all Madagascan species fell in the Madagascan clade. These results are in line with the tree obtained using Bayesian inference.

**Fig 4:**
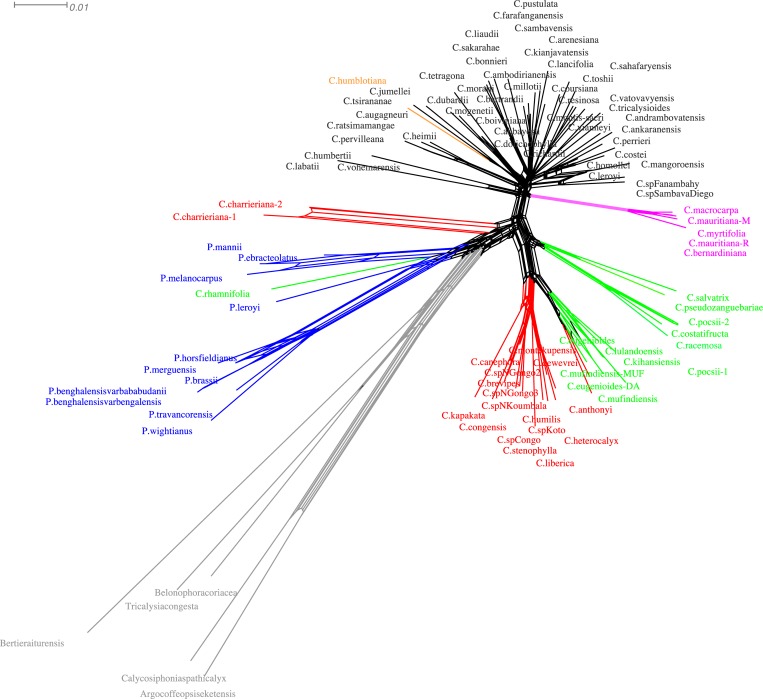
Nuclear phylogenetic network representation. The NeighborNet method of the Maximum likelihood nuclear phylogeny of coffee trees presented in Fig 3 was used to obtain the phylogenetic network.

The phylogenetic network study, for its part, tended to indicate that horizontal transfers of genetic material have been frequent between the West and Central African species *P*. *ebracteolatus*, *P*. *melanocarpus*, and *P*. *mannii*, and at least as much as in the Asian *Psilanthus* branch (*P*. *horsfieldianus*, *P*. *brassii*, *P*. *merguensis*, *P*. *benghalensis* var bengalensis, *P*. *benghalensis* var *bababudanii*, *P*. *travancorensis*, and *P*. *wightianus*), (Fig 4).

### Maternal coffee phylogenenetic relationships

The alignment of the whole Cp genomes in this study led to 157,020 selected sites, including 152,270 complete (no gaps, no N), 2,877 variable (1.9% of complete), and 1,250 informative (0.8% of complete) sites. The base composition was almost the same everywhere: 30.8% adenine, 19.1% cytosine, 18.5% guanine, and finally 31.6% thymine, while these rates varied by less than 0.1% from one genome to another. Focusing on the observed changes, the mean transition/transversion ratio was equal to 1.01 with 1,310 transitions and 1,296 transversions. The investigation of variable sites has been deepened by the polymorphism study, indicating a total of 2,606 single nucleotide polymorphisms (SNPs), 163 indels, and 328 gaps. The average size of gaps was 13.6 nucleotides, while the standard deviation of these sizes was equal to 25.7, revealing the great variability in these gaps. Reduced to the length of the alignment, we obtained a percentage of SNPs, indels, and gaps respectively equal to 1.6%, 0.1%, and 2.84%.

**Fig 5:**
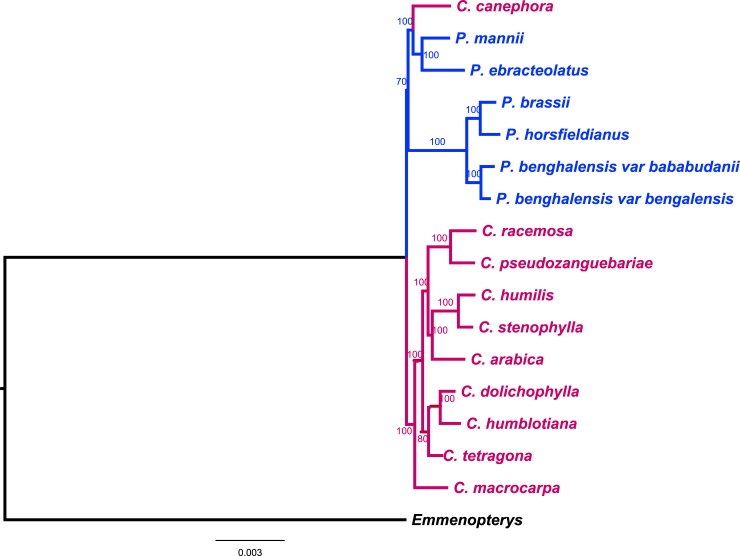
Maternal phylogenetic relationships. The ML phylogenetic tree was constructed based on a set of 16 coffee trees representatives of the main geographic clades, and whole chloroplast sequences study using *Emmenopterys henryi* as outgroup. *Psilanthus* (in blue) and *Coffea* (in red) species clustered into two well-supported clades with the exception of *C*. *canephora* belonging to the *Psilanthus* clade.

The chloroplast phylogeny depicted in Fig 5 agreed with and reinforced the nuclear one. With the exception of *C*. *canephora*, two well-separated clades were obtained, one constituted with *Psilanthus* species only, and the second with *Coffea*. The change of outgroup (from *Emmenopterys henryi* to *Solanum lycopersicum*) did not change the topology of the tree, but decreased the support upstream of the clade containing the *Psilanthus* genus. In this tree, *C*. *canephora* does not belong to the *Coffea* clade but is close to the African *Psilanthus* (*P*. *ebracteolatus* and *P*. *mannii*). In the nuclear tree, however, *C*. *canephora* does not group with *Psilanthus* but with other *Coffea* from its native area (i.e., west and central Africa). This unexpected position of *C*. *canephora* in the maternal phylogeny was independent of the chosen outgroup. Each time, the same topology was obtained, with *C*. *canephora* supports close to 90.

### Gene content evolution over time

**Fig 6:**
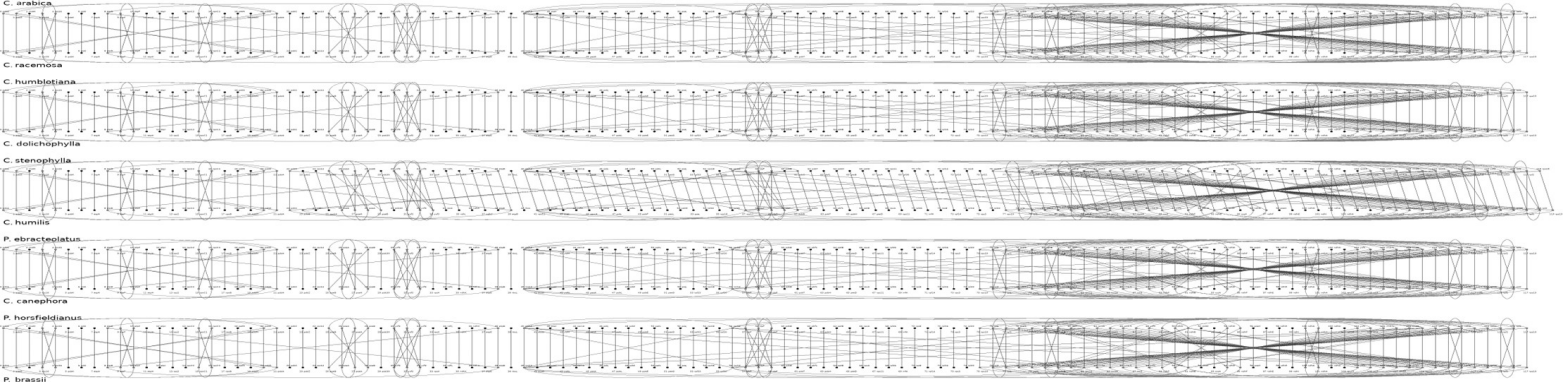
Cp genome gene contents for some pairwise comparisons based on GeSeq annotations. Each of the five images compares the two genomes of sister species in phylogeny. Each genome is represented as a dotted line, each dot being a gene (the dot is labeled by its name). A red curve indicates paralogy between genes inside a given genome, while a blue line is for gene orthology between two distinct genomes.

As stated previously, the complete Cp genomes were all annotated using GeSeq. The Cp genomes of all studied coffees consisted of 84 genes in the same ordering. However, in the pairwise comparison with *C*. *stenophylla*, *C*. *humilis* seemed to have an additional *spbB* gene at position 2 (Fig 6). To determine if this ordering difference can be attributed to an annotation error, the gene sequence was blasted against the *C*. *stenophylla* Cp genome. Only two thirds of the sequence was found (at position 32,109) suggesting that *spbB* is incomplete in *C*. *stenophylla* and probably not functional, explaining why GeSeq failed to detect it.

**Fig 7:**
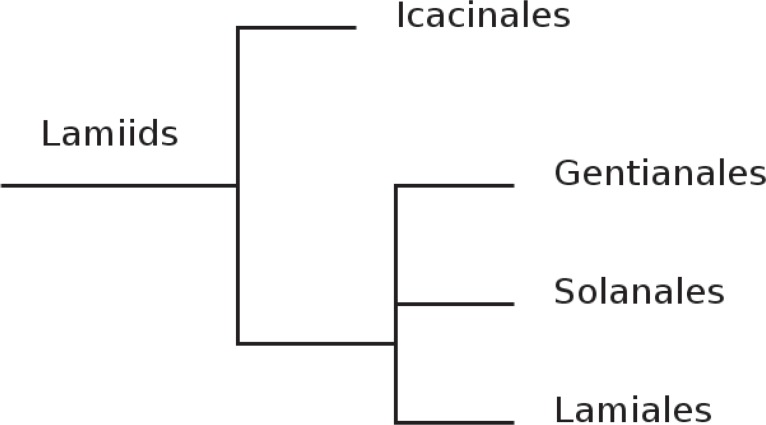
Gentianales and its neighboring orders phylogenetic relationships. The tree is based on the taxonomy and data available on the NCBI website.

Coffee gene content was also exactly the same as that for all Cp genomes available for Gentianales. This content began to differ at the Lamiid level (Fig 7). Noteworthy, the same gene content as that found in *Coffea* was present at least in one species of each order in this tree.

Compared to the 92 genes constituting the plant chloroplast pan genome identified among more than 2,000 available Cp genome sequences using GeSeq (unpublished data), eight genes were always absent in coffee trees, namely: *CHLB* (known to be required for light-independent chlorophyll accumulation in *Chlamydomonas reinhardtii* [41]), *CHLL*, *CHLN*, *CYSA*, *CYST*, *MBPX*, *PSAM*, and *RPL21*.

### Investigation of mutations over time

**Fig 8:**
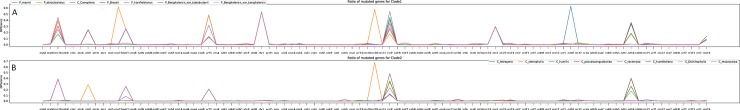
Gene mutation rates per species among each clade. The gene mutation rate between a species and its ancestor for a given gene is equal to the Levenshtein distance between the translated gene sequences of the specie and the ancestor divided by the length of their alignment. (A) Gene mutation rates between the species in clade 1 (*Psilanthus*) and the reconstructed ancestor of the clade. (B) Gene mutation rates between the species in clade 2 (*Coffea*) and the reconstructed ancestor of the clade.

We reconstructed the ancestral Cp genomes, to investigate which genes have mutated over time. As we needed "cousins" to solve the problem cases, we reconstructed the ancestors of the two clades separately (*Coffea* and *Psilanthus*), and within each clade, genes of each species were compared to the reconstructed ancestor. The mutation ratio obtained relative to the last common ancestor of the clades were depicted in Fig 8A and 8B. As can be seen, most of the genes have not evolved over time, while mutations that occurred were not concentrated in one particular location of the genome. Furthermore, *Psilanthus* (Clade 1) accumulated more mutations than *Coffea* (Clade 2). Clade 1 had two particular genes, namely *rbcL* and *rps3*, that mutated at the separation of the two clades as all species of Clade 1 have the same copy of these genes, which were different from the ancestral genome.

Other genes appeared to have a clear propensity to mutate, either in a single clade or in both. This was the case for *rps16* and *ycf3* (mainly in Clade 1), *clpP* and *ndhA* (in both clades). Genes have been ordered according to their variability in decreasing order) (Table 2).

**Table 2:** Number of species in which one or several genes gene are different from the ancestral version. Genes have been ordered according to their variability, from highly to less variable and according to the number of species in which these genes are different from the ancestral version. For instance, all the species of Clade 1 (7) have *rpl16* and *rps16* different from their ancestor.

number of species	Clade 1 (*Psilanthus*)	Clade 2 (*Coffea*)
9		matK, rpoC2, rpoC1, rpoB, rbcL, clpP, psbB, rps3, ycf1
8		rps16, ccsA, ndhA, ndhF, ndhD
7	matK, rps16, atpF, rpoC2, rpoC1, psaA, ycf3, rbcL, accD, clpP, rpoA, rpl16, rps3, ycf1, ndhF, ccsA, ndhD, ndhA, rps19	accD, ycf2, ndhH
6	rpoB, rps4, psbB, rps8, petA,	rps8, rpl22, ycf3, psaA
	rpl20, ycf2	
5	atpA, cemA, rps2, ndhC, psaB	atpF, psbC, rpoA, rpl2, rpl32
4	rpl22, rps18, ndhI, psbT, petD, rpl14, rpl2, rps12	rps14, rps4, atpA, atpB, rps19
3	psbD, psbC, rps15, ycf4, ndhB	rps18, atpI, rpl20, petD, rpl36, ndhI, psaB
2	atpI, ndhK, ndhG, rpl32, ndhE, rps11, ndhH	psbA, ndhK, petA, rpl33, rpl14, ndhJ, ycf4, rpl16, rps12, ndhG, psbD, petG, ndhB
1	psbA, rpl33, atpE, atpB, psbZ, ndhJ, rpl23	psbK, ndhC, rps15, rpl23, atpE, cemA, rps11, psaJ

**Fig 9:**
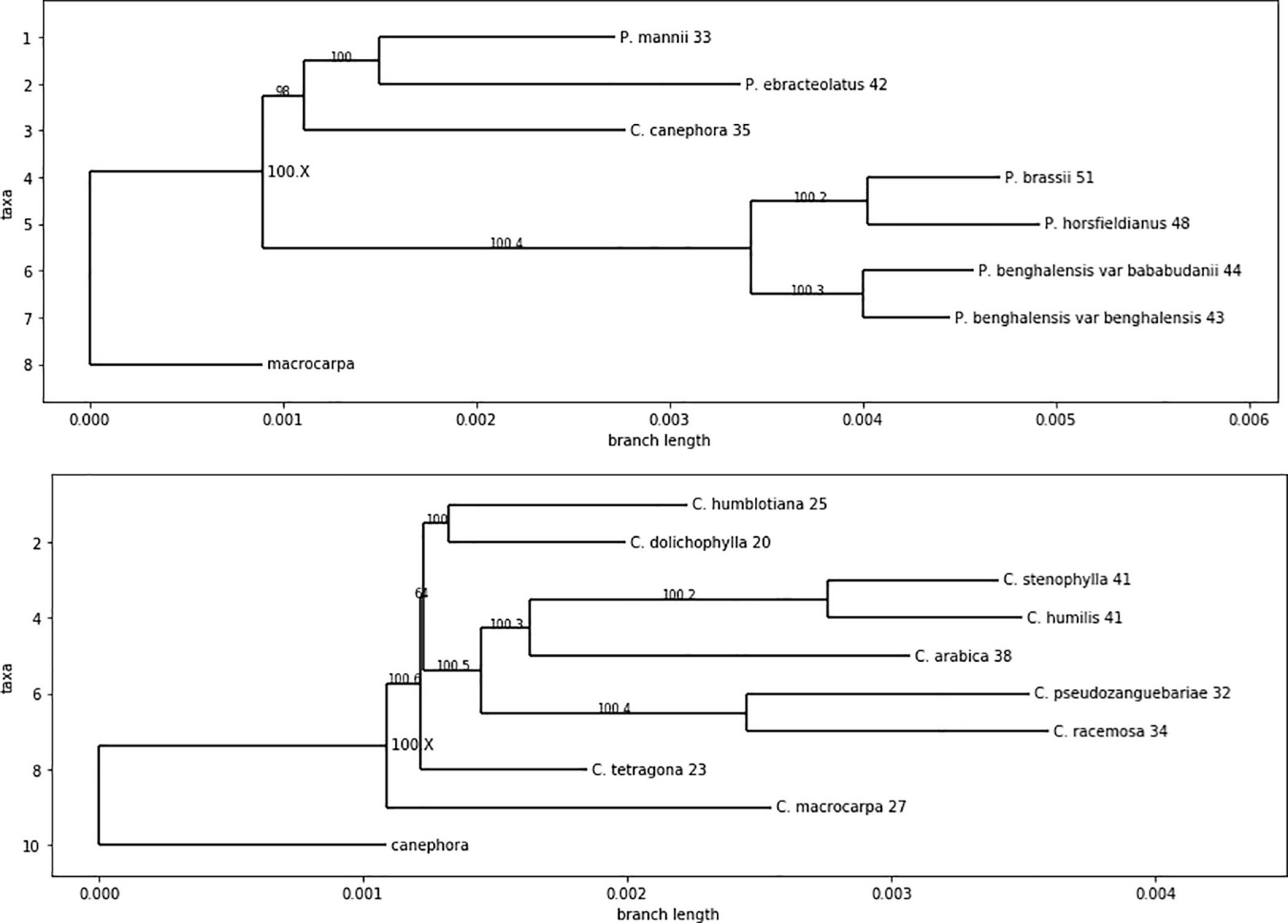
Phylogenetic tree showing the number of mutated genes per species. Phylogenetic tree with the number of mutated genes per species when compared to the ancestor (numbers next to the species names) of the clade (A)- Clade 1 (*Psilanthus*); (B)- Clade 2 (*Coffea*). Branch values are bootstraps according to the maximum likelihood phylogenetic reconstruction. A suffix has been added to provide unique names to ancestral nodes.

It can be noticed that some genes had a strong tendency to evolve in relation to their ancestors, regardless of the context, when those with only one or two variants in current species differed according to the clade. This was the case for genes *ycf1*, *ycf2*, and *matK*, the latter is therefore justifiably used in phylogenetic studies, like in the *Lilium* case. Let note that the difference of evolution of genes *rbcL* and *matK* may explain incompatible molecular phylogenies in *Lilium* [42, 43]). Conversely, some genes have varied less from one clade to another, which was the case of *rps12*. In the Fig 9A and 9B, we display the clade trees and, next to the species names, we show the number of mutated genes in each species in relation to the ancestor. A large variability can be reported, ranging from 20 mutated genes (*C*. *dolicophylla*) to 51 (*P*. *brassii*). On the other hand, each subclade had, on average, a number of mutated genes of the same order of magnitude, which was consistent with the principle of evolution over time.

Focusing on highly mutated genes per species (less than 95% of similarity with the last common ancestor of coffee trees), for Clade 1, we found: *C*. *canephora*: *rps16*, *ycf3*, *rbcL*, *clpP*, *rps3*, *ndhA*, and *rps19; P*. *benghalensis* var bababudanii: *rps16*, *atpF*, *ycf3*, *rbcL*, *clpP*, *rps3n ndhA*, and *rps19;*. *P*. *benghalensis v*ar benghalensis: *rps16*, *ycf3*, *rbcL*, *clpP*, *rps3*, *and rps19; P*. *brassii*: *rps16*, *atpF*, *rpoC1*, *ycf3*, *rbcL*, *clpP*, *rps3*, *ndhA*, and *rps19; P*. *ebracteolatus*: *rps16*, *rpoC2*, *rpoC1*, *ycf3*, *rbcL*, *rpl20*, *clpP*, *rps3*, *ndhA*, and *rps19; P*. *horsfieldianus*: *rps16*, *atpF*, *rpoC1*, *ycf3*, *rbcL*, *clpP*, *rps3*, and *rps19;*. *P*. *mannii*: *rps16*, *rpoC1*, *ycf3*, *rbcL*, *clpP*, *rps3*, *ndhF*, *ndhA*, and *rps19*. The highly mutated genes per specie for Clade 2 were: *C*. *tetragona*: *rpoC1*, *clpP*, and *ndhA; C*. *stenophylla*: *atpF*, *rpl20*, *clpP*, and *ndhA; C*. *humilis*: *rps16*, *rpoC1*, *clpP*, and *ndhA; C*. *pseudozanguebariae*: *rps16*, *ycf3*, and *clpP; C*. *racemosa*: *rps16*, *rpoC1*, *ycf3*, and *clpP; C*. *humblotiana*: *rps16*, *rpoC1*, *clpP*, and *ndhA; C*. *dolichophylla*: *rps16*, *rpoC1*, and *clpP; C*. *macrocarpa*: *clpP* and *ndhA*.

## Discussion

In this study, we compared annotation results produced with Dogma or with the more recently developed, GeSeq [14]. A careful state-of-the-art study has emphasized that, most of the time, Dogma has been widely used for gene prediction in chloroplasts [25, 12, 44]), frequently taking the annotations produced at face value and not asking too many questions about the quality of the results. Indeed, until recently, it was the only tool specific to chloroplast genomes, which explains its success and its almost exclusive use for the annotation of such genomes. However, despite Dogma being a relatively old tool (2000–2004) and being considered out-of-date, it was used for the chloroplast annotation of *C*. *arabica* [25] and more recently for the chloroplast of *C*. *canephora* [12], currently the only available complete annotated genomes for *Coffea* and for seeds plants [8]. So, it was important to determine if Dogma quality could, at present, still be considered to be adequate. To evaluate this, criteria for evaluation based on our previous research were used [9, 45], including the simplicity of the process, and the ability for the annotation tool to recover genes from a collection of plant chloroplasts whose annotation goes back a long time (and as a result have been well curated manually, like *Nicotiana tabacum*, *Olea europaea L*., *Solanum lycopersicum*, or even *Ginkgo biloba*).

Our results were in line with the opinion of Dogma developers: quoting their website, "while it remains useful to many, it is not under active development, will not be updated, and is unsupported, so please manage your expectations accordingly." According to our own benchmarks, its database and technique is now too old and out-of-date, multiplying false positives and negatives in gene detection. Conversely, a careful investigation of the annotations performed by GeSeq revealed less differences in gene content between any pair of chloroplast genomes, which was in line with the close relationship of the species being considered.

Outside Geseq and Dogma, other annotation tools are available, such as PLANN (Plastome Annotator) [46], CpGAVAS (Chloroplast Genome Annotation, Visualization, Analysis and GenBank Submission) [13], Verdant [47], CGAP (Chloroplast Genome Analysis Platform) [48] and AGORA [15]. Alternative organellar annotation tools to GeSeq have been investigated too (https://chlorobox.mpimp-golm.mpg.de/Alternative-Tools.html), but either they can only annotate one genome at a time (Agora, CpGavas), or they no longer work (Mfannot, MITOFY), or they focus only on mitochondria (MitoAnnotator, MITOS, or MOSAS). GeSeq was also the most efficient procedure to annotate the 2,112 chloroplast genomes under consideration [unpublished data].

The use of the complete Cp sequences permitted the generation of a maternal coffee phylogeny and showed two well-supported main clades, one exclusively constituted by *Coffea* species, the other by all *Psilanthus* plus *Coffea canephora*. The particular position of *C*. *canephora*, (Fig 5), contrary to its position into the nuclear phylogenetic tree (Fig 3) was intriguing. To understand the significance and the origin of its chloroplast relationships, a sampling and sequencing of *C*. *canephora* in Africa (from Guinea to West of Tanzania and from Centrafrican Republic to North Angola) and closely related species is now necessary.

The same gene content and ordering for all coffee genomes was found. Therefore, 84 genes were identified which is slightly higher than the average of all the 2,112 plant genomes analyzed previously [unpublished data] (81.9 genes). Moreover, the same gene content as that found in *Coffea* is present in Gentianales and at least in one species of each order investigated (Fig 6) arguing for an even older origin of this form of chloroplast, based upon gene content.

Gene losses were already reported in Poaceae [49]. The eight missing genes in coffees are intriguing. Are they really absent, that means eliminated over time and if so, when? Did they integrate the nuclear genomes and when? In this case, are they always active or not? The availability of nuclear raw data for a large number of *Coffea* and *Psilanthus* species should help in resolving this question.

Investigation of mutations over time revealed some interesting features, such as the particular case of the *rbcL* gene. The chloroplast rbcL gene encodes the large subunit of Ribulose-l,5-bisphosphate carboxylase/oxygenase (Rubisco, involved in plant photosynthesis) and plays a major role in carbon assimilation. Studies on Brassicaceae [50], *Rheum* [51] showed positive selection in *rbcL* gene assuming that adaptation of species to different habitats can be correlated with *rbcL* evolution.

Although *rbcL* gene would evolve rapidly, in coffees, this gene had a fairly binary evolution over time, presenting only two orthologs corresponding to the two clades identified here (*Psilanthus* and *Coffea*). The phylogenetic information it carries was therefore relatively poor, at least in the case of the species of interest to us. This might raise the question of the relevance of its frequent use for molecular phylogeny studies, such as for *Lilium* and allied genera. As well, the difference in rate of evolution of *rbcL* and *matK* might explain the incompatible molecular phylogenies of *Lilium* [42, 43]. Other genes appeared to have a clear propensity to mutate, either in a single clade or in both. One possibility is that these genes could be of relative importance, and are therefore not very constrained keeping in mind that synonymous and non synonymous mutations have not the same consequence in terms of selective pressure and gene evolution.

By its gene mutation rates, *C*. *canephora* is more similar to that observed for Clade 1 (*Psilanthus)* than for Clade 2 (*Coffea*). Finally, both by its position in the maternal phylogenetic tree and by its gene mutation rates, *C*. *canephora* appeared not really different from other *Psilanthus*.

## Conclusion and future work

The analyses of a set of coffee species representatives of the main geographic clades permitted us to develop the best methodology to investigate ancestral reconstruction and Cp genome evolution over time. Investigation of annotation tools revealed some biases in their utilization and the conclusions that can be drawn from them. In this context, most of the annotated chloroplast genomes available on the NCBI are based on a tool that was, at the time, the best available, but which is now outdated and gives too many false positives and negatives. Our study on the coffee case shows that previously released annotated chloroplasts must be disregarded together with the conclusions that can be drawn from them, while the use of up-to-date annotation methods appears more satisfactory.

The molecular phylogeny based on complete Cp genome alignment supports two main clades; one including only *Coffea* species and the other all *Psilanthus* plus one *Coffea*. The study of a wider set of species will help determine whether this feature is unique or not. Currently, when genes are compared with the ancestor of the two clades, the orthologs are considered as they are, without separation of the introns and exons. In future work, it would be interesting to go into more detail, by calculating the mutation rate of the coding and non-coding parts. This rate of mutation of the genes could be compared to that of the intergenic regions, at the nucleotide and acid-amino levels, which would also allow the silent mutation ratio to be seen. We could look at whether substitutions/indels occurred more frequently in exons (or introns, or both indifferently), and we could try to understand how mutations accumulate at random or not, and at what rate? Another piece of information that may be interesting to compare is the G+C rate. Similarly, it could be interesting to see whether mutations are more frequent at the breaking points upstream and downstream of the quadripartite structure, focusing in particular on what is happening between *C*.*arabica* on the one hand, and *C*. *canephora* (the paternal parent of *C*. *arabica*) and *C*. *eugenioides* (the maternal parent of *C*. *arabica*) on the other hand. Indeed, as chloroplasts are inherited maternally and *C*. *arabica* has the chloroplast of *C*. *eugenioides*, this latter should not be placed with *C*. *canephora* but with *C*. *arabica* (so within the *Coffea* clade, as expected). In terms of gene content evolution, we wonder if the eight missing Cp genes in the coffees have been transmitted to the nuclear genome, and if transfers have taken place in the other direction over time. We need to know when, in the tree of life, gene(s) disappeared among the ancestors of *C*. *arabica*, and if the order of genes has changed over time. The function of the missing genes in particular could be examined, in order to determine whether some of them are of special importance.

## Supporting information

S1 Table Coffee species considered in the nuclear phylogeny study. Additional samples to those used in Hamon et al. (2017) are marked by an asterik.
(XLSX)
